# CD34 and Ki-67 Immunoexpression in Periapical Granulomas: Implications for Angiogenesis and Cellular Proliferation

**DOI:** 10.3390/diagnostics14212446

**Published:** 2024-10-31

**Authors:** Ciprian Roi, Mircea Riviș, Alexandra Roi, Marius Raica, Raluca Amalia Ceaușu, Alexandru Cătălin Motofelea, Pușa Nela Gaje

**Affiliations:** 1 Department of Anesthesiology and Oral Surgery, Multidisciplinary Center for Research, Evaluation, Diagnosis and Therapies in Oral Medicine, “Victor Babeș” University of Medicine and Pharmacy, Eftimie Murgu Sq. no. 2, 300041 Timisoara, Romania; ciprian.roi@umft.ro (C.R.); rivis.mircea@umft.ro (M.R.); 2 Department of Oral Pathology, Multidisciplinary Center for Research, Evaluation, Diagnosis and Therapies in Oral Medicine, “Victor Babeș” University of Medicine and Pharmacy, Eftimie Murgu Sq. no. 2, 300041 Timisoara, Romania; 3 Department of Microscopic Morphology/Histology, Angiogenesis Research Center, “Victor Babeș” University of Medicine and Pharmacy, 300041 Timisoara, Romania; marius.raica@umft.ro (M.R.); ra.ceausu@umft.ro (R.A.C.); gaje.nela@umft.ro (P.N.G.); 4 Department of Internal Medicine, Faculty of Medicine, “Victor Babeș” University of Medicine and Pharmacy, 300041 Timisoara, Romania; alexandru.motofelea@umft.ro

**Keywords:** CD34, Ki-67, periapical granuloma, granulation tissue, cell proliferation, angiogenesis

## Abstract

Background/Objectives: The main mechanism of the formation of granulation tissue is the progression of an infection from the tooth to the periapical bone. At this level, the immune system tries to localize and annihilate the microorganism’s injury. Ki-67 is a protein directly associated with the cell proliferation rate, while CD34 is a biomarker involved in angiogenesis, and studies suggest that they both have a positive correlation with the intensity of the local inflammatory infiltrate. This study will determine the immunoexpression of CD34 and Ki-67 in periapical granulomas and assess their impact on the growth and development of this tissue, as well as consider their roles in the proliferative process and aggressiveness of evolution. Methods: In the present study, 35 periapical granulomas obtained after a tooth extraction were included. The specimens were analyzed via histopathology and immunohistochemistry. Results: A positive reaction for the Ki-67 antibody was observed in 32 (86.5%) of the 35 periapical granuloma cases included in our study. We identified the overexpression of Ki-67 and CD34 and further calculated the Ki-67 index to evaluate and correlate the proliferation potential and angiogenesis with regard to the presence of an inflammatory infiltrate. Conclusions: These findings suggest that the persistence of an inflammatory environment directly influences Ki-67 and CD34 expression, sustaining the proliferative capacity of cells and abnormal angiogenesis. This study is the first to evaluate the presence of the CD34+ and Ki-67+ proliferating vessels in periapical granulomas.

## 1. Introduction

One frequently encountered pathology affecting the alveolar bone is periapical periodontitis, a reaction caused by the invasion and proliferation of microorganisms due to untreated pulp necrosis [1]. The interaction between these microorganisms and the immune system’s response to their action determines periapical periodontitis development. The persistence of the infection determines local bone resorption, followed by the replacement of bone tissue with granulation tissue [2]. It is estimated that, worldwide, 52% of adults have at least one periapical periodontitis-affected tooth [3]. Nair et al. classified periapical radiolucency into apical abscesses, acute apical periodontitis, apical cysts, and chronic apical periodontitis, identified as periapical granulomas. The World Health Organization does not include the term “periapical lesion” in their classification [4,5].

These modifications can also be explained by an alteration in the eubiosis of an oral cavity. In the past, only the concept of probiotics and their important roles were known; nowadays, novel products, such as paraprobiotics (tyndallized probiotics) and postbiotics, have been created, and they can play a protective role in the occurrence of periapical pathologies [6].

Granulation tissue, as a precursor of periapical granulomas, predominantly includes the lymphocytes, plasma cells, macrophages, and mast cells that influence the development of periapical granulomas [7]. Nevertheless, the presence of mast cells in inflammatory granulomas highlights their role in this inflammatory process [8].

A histological analysis of periapical granulomas shows a strong angiogenesis process affecting the newly formed vessels, and that significant immune cell infiltration secretes the growth factors and cytokines that affect cells’ continued proliferation and migration [9].

The presence of an inflammatory infiltrate and the activation of neo-angiogenesis have direct implications for the continuous development of periapical granulomas. As the inflammatory stage continues, it triggers the release of multiple angiogenic factors that directly act upon different cells [10]. Angiogenesis in oral pathologies potentiates progression and sustains inflammation, and it is linked to an unfavorable prognosis. During its progress, it determines the formation of new blood vessels through the proliferation and migration of endothelial cells. The formation of new vessels is accompanied by the introduction, among oxygen and nutrients, of pro-inflammatory cells into pathological tissue [11].

Since different pathological mechanisms and changes are involved in periapical granulomas’ occurrence, persistence, and progression, multiple markers linked to these could be targeted to evaluate this pathological lesion. Studies have focused on evaluating CD34 to assess the presence of endothelial cells and the progress of angiogenesis in pathological tissue [12,13].

CD34 is a macromolecular transmembrane sialomucin protein, and it was first discovered in human hematopoietic progenitor cells [14]. Besides the bloodstream, it is found in vascular endothelial cells, keratinocytes, fibrocytes, interstitial cells, and epithelial progenitors [15]. The presence of the CD34 marker has significant involvement in angiogenesis, and studies also suggest that it has a positive correlation with the intensity of the local inflammatory infiltrate [16].

Ki-67 is a protein directly associated with the cell proliferation rate. The presence of this protein has been reported at all evolution stages of cells [17], making it a viable marker responsible for the growth rates of cells [18]. To outline this aspect, past research identified highly expressed Ki-67 in all phases of a cell cycle, being outlined in G1, S, G2, and mitosis. One important aspect was the absence of Ki-67 expression in the resting cell stage (G0) [19].

To assess the level of Ki-67, the percentage of cells labeled with a representative antibody for Ki-67 must be quantified [20]. Through the evaluation of Ki-67 and its presence in odontogenic inflammatory lesions, important information regarding the proliferative potential of the cells and the recurrence rate can be obtained [20]. Nevertheless, several studies have identified the presence of high levels of Ki-67, showing that it is a potential biomarker for acknowledging the aggressive behavior of odontogenic cystic lesions [21]. In addition, a positive Ki-67 reaction has been identified as a predictor of proliferation as a result of a chronic inflammatory environment [22]. Several studies have discussed the effects of higher levels of Ki-67 on the aggressive behavior of these granulomas as a consequence of the continuous activation of the inflammatory cells by the existing microorganisms [23].

In the development of periapical granulomas, the inflammatory environment plays a key role, maintaining the proper conditions and cell populations required for the further progression of this pathological entity. Based on the involvement of several bacterial populations that trigger cytokine and growth factor production, the cell proliferation rate is directly influenced and sustained. The release of cytokines will determine the rate of increase in cellular stress and the increased immunoexpression of inflammatory markers, such as Ki-67 [24] and CD34 [16]. This aspect appears to directly influence the proliferation rate of pathological tissue, as well as further immunopathological interactions.

The present study aims to identify and evaluate the immunoexpression of CD34 and Ki-67 in periapical granulomas, as well as determine their influence on the development and progress of this type of tissue, considering their influence on the proliferative process and aggressiveness of evolution. By understanding this pathogenesis, cellular and molecular interactions, and the existing changes related to periapical granulomas, treatment approaches can be optimized.

## 2. Materials and Methods

This cross-sectional study was conducted during September 2022–March 2024 and approved by the Ethics Committee of “Victor Babeș” University of Medicine and Pharmacy Timișoara (no. 39/2022). All the patients included in the present study signed an informed consent form that followed the guidelines of the Declaration of Helsinki.

### 2.1. Patients

The inclusion and exclusion criteria were as follows:

The inclusion criteria:

- Age: 18–70 years;
- Both males and females;
- Teeth with an indication of exodontia due to the impossibility of restorative treatment and presence of a periapical granuloma;
- Non-vital teeth;
- Teeth without endodontic treatment.

The exclusion criteria:

- Minor patients;
- Patients with cervico-facial or/and oral cancers;
- Patients with mucositis;
- Patients with altered general conditions: acute leukemia, recent myocardial infarction, or a stroke in the last 6 months;
- Patients undergoing drug treatment for bone pathologies (e.g., bisphosphonates).

Based on the inclusion and exclusion criteria, 35 patients were included in this study, namely 17 females and 18 males, with an age range from 24 to 72 years. Regarding smoking status, 25 patients were non-smokers and 10 were smokers.

### 2.2. Clinical Assessment and Granuloma Harvesting

Patients with preoperative periapical radiological radio transparency specific to an odontogenic granuloma and clinically evident or suspected periapical lesions involving non-vital teeth (as determined by an electric pulp tester) were included in this study. In addition, only teeth with no clinical indications of restauration were included. All the patients underwent a standardized preoperative orthopantomogram radiography. All radiographic films were exposed and processed under similar conditions. A radiographic evaluation was performed based on the Periapical Index (PAI) scoring system [24].

After tooth exodontia, the periapical granulomas were removed via the curettage of the root socket. In our study, 29 patients had one tooth extracted, 5 patients had two teeth extracted and 1 patient had three teeth extracted. In this study, 22 teeth were maxillary teeth and 13 were mandibular teeth.

After socket curettage, the periapical granulomas were immersed in formalin, and a fixation of the specimens was carried out with 10% buffered formalin for 48–72 h.

### 2.3. Primary Probe Processing

We performed an observational study on the paraffin-embedded periapical tissue slices of the 35 periapical granuloma specimens processed according to a standard histology technique. The probes were cleaned, dried, clarified, and imbedded in paraffin. The Thermo Shandon standardized inclusion automat (Thermo Fisher Scientific Inc., Aren-dalsvägen 16–418 78 Gothenburg, Sweden) was utilized for the inclusion stage. The Shandom ME microtome was used to perform sectioning. Two slices, each measuring around 3 to 5 μm in thickness, were cut from each paraffin block. Hematoxylin–eosin staining of one set of sections was performed using the Leica automatic system on a regular basis to verify the clinical diagnosis.

### 2.4. Immunohistochemistry

For all the cases included in this study, double immunostaining was performed through a fully automated and standardized procedure for all the cases, using a Leica Bond-Max auto-stainer (Leica Biosystems, Newcastle upon Tyne, UK). Paraffin sections were treated for 20 min with a Bond Epitope Retrieval 2 solution (Leica Biosystems, Newcastle Ltd., Newcastle Upon Tyne, UK). Endogenous peroxidase was blocked with 3% hydrogen peroxide for 5 min. Then, the sections were incubated for 30 min with the CD34 primary antibody (Leica Bond, RTU, clone QBEnd/10, Leica Biosystems Nussloch GmbH, Nußloch, Germany).

For visualization, we used the Bond Polymer Refine Detection System, including the secondary antibody (8 min) and the polymer, with an 8 min incubation time. After peroxidase blocking, we applied the second Ki-67 antibody (Leica Bond, RTU, clone MM1). The Bond Polymer Refine Red detection system containing 3,3-diamino-benzidine dihydrochloride and hematoxylin was used for visualization. Stained sections were permanently mounted with Canada balsam.

### 2.5. Microscopic Evaluation and Image Analysis

Sections stained morphologically with hematoxylin–eosin and immunohistochemically via CD34-Ki-67 double immunostaining were analyzed using the Zeiss Axiocam 506 (Jena, Germany) and Nikon AY260 microscopes (Nikon Europe B.V., Amstelveen, The Netherlands). Both microscopes are equipped with a real-time imaging system and software for the digital analysis of microscopic images.

The assessment of microvascular density was performed according to the modified Weidner method [25]. Three microscopic fields with maximum vascular densities were chosen, and they had a ×400 magnification. The arithmetic mean represented the final result.

Ki-67 was assessed via the original semi-automated method [26] on the immunohistochemically stained sections. Each slide was initially examined with a ×100 magnification, and the areas with the highest densities of Ki-67-positive nuclei were selected. The Ki-67 proliferation index was calculated using the digital images captured with a ×400 magnification. The percentage of positive cells expressing Ki-67 was calculated, and the expression level was evaluated using a scoring system developed by the American Association for the Study of Cell Biology [27,28]. Applying this approach gave a score for the proportion of positively immunostained cells [(absent 1%), (mild 1–10%), (moderate > 10–30%) and (strong > 30%)] ranging from 0 to 3.

### 2.6. Statistical Analysis

The continuous variables following a normal distribution were presented as means with standard deviations (SDs), while non-normally distributed data were presented as medians with interquartile ranges (IQRs). The distribution’s normality was evaluated using the Shapiro–Wilk test. The differences among the groups for the normally distributed continuous data were assessed using the Welch’s *t*-test for two groups or the ANOVA for more than two groups. Post hoc analyses, when necessary, were performed using the Bonferroni correction to adjust for multiple comparisons. For the non-normally distributed continuous data, the Mann–Whitney U test and the Wilcoxon signed rank test were used for two-group comparisons, while the Kruskal–Wallis test was applied for comparisons involving three or more groups. The false discovery rate was applied to adjust for multiple comparisons in the Mann–Whitney U test, Wilcoxon signed rank test, and Kruskal–Wallis test. The categorical data were analyzed using the χ^2^ test or Fisher’s exact test, particularly when the expected cell counts were below five. The categorical data were reported as frequencies (*n*) and percentages (%). A prior power analysis was performed with at least 80% statistical power and a 95% confidence interval. All statistical analyses were performed using R Studio version 3.6.0, using the packages stats, dplyr, coin, multcomp, and pwr. For the logistic regression model analyzing the Ki-67 score, model fit measures included deviance, AIC, and McFadden’s R^2^. The deviance was 62.8, the AIC was 119, and McFadden’s R^2^ was 0.330.

## 3. Results

In the present study, 35 periapical granulomas obtained after the extraction of teeth were included. The patients were enrolled, and the details related to demographic data and potential associated risk factors were further analyzed to identify the potential correlations. The histopathological examination of the samples involved describing the existing cellular population and evaluating CD34 and Ki-67 expression.

The median age of the patients included in this study was 43 years old, with an interquartile range (IQR) of 36 to 50 years. Out of the 35 patients, 17 (49%) were female. Ten patients (27%) were smokers, and among the smokers, the median daily cigarette consumption was 20 cigarettes, with an IQR of 16.3 to 20 cigarettes per day (Table 1).

For the average proliferating vessels, significant differences were observed between the Ki-67 score groups, with the highest values being found in the >30 group (mean = 8.0), followed by the 20–30 group (mean = 6.0). The statistical test had a significant effect, with *p* < 0.011.

The connective tissue vessels had a consistent median across the groups, with the highest value recorded in the >30 group (median = 15.0); however, the differences were not significant (*p* = 0.821).

The results indicate that the majority of patients across all the Ki-67 score groups had one tooth extracted, with 83.8% of patients placed in this category. Regarding sex distribution, females comprised 49% of the total sample, and they were more highly represented in the 10–20 Ki-67 group (100%). Males made up 51% of the sample, and all the patients in the >10 group were male.

**Figure 1:**
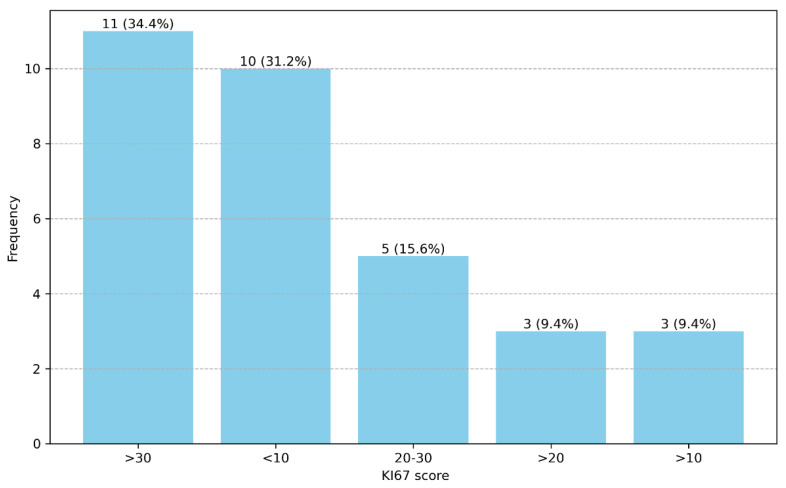
The distribution of the Ki-67 scores.

Figure 1 shows a bimodal distribution, with 34.4% of the subjects having Ki-67 scores greater than 30, 31.2% having scores less than 10, and smaller proportions falling within the intermediate ranges.

In the logistic regression model for the Ki-67 score groups, significant predictors included “Proliferant vessels” for the <10 vs. 20–30 comparison (estimate = −35.637, *p* < 0.001), and “Connective tissue vessels” for the >20 vs. 20–30 comparison (estimate = −8.333, *p* = 0.048). The other predictors did not show significant effects.

### 3.1. Histopathological Analysis

The periapical granuloma tissues were analyzed based on the diagnostic criteria of Omoregie et al. [29,30].

**Figure 2:**
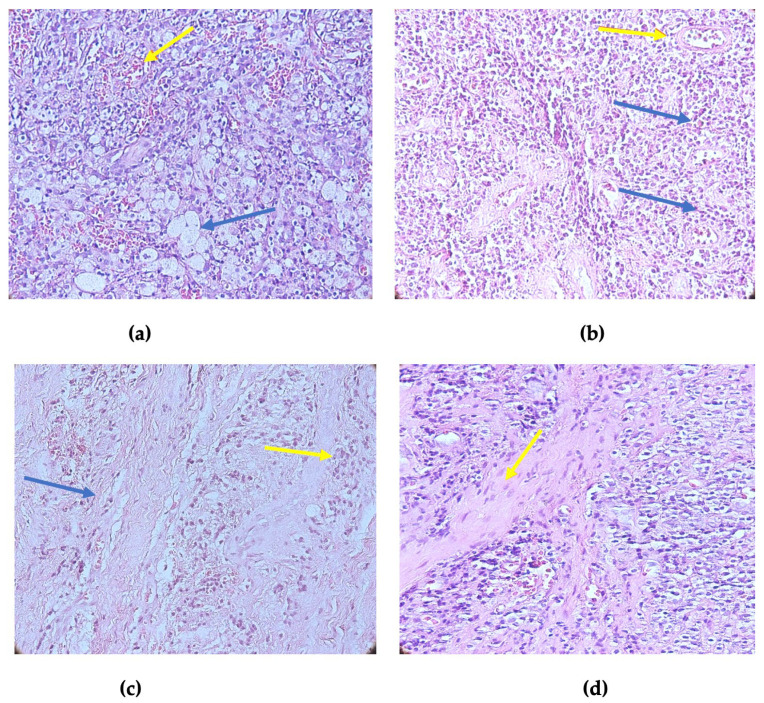
Histopathological aspects of periapical granulomas: (**a**) early periapical granuloma with numerous foamy macrophages (blue arrow) and blood vessels (yellow arrow); (**b**) intermediate periapical granuloma with mixed inflammatory infiltrate consisting of lymphocyte plasma cells and macrophages (blue arrows) and blood vessels (yellow arrow); (**c**) late periapical granuloma with rich connective stroma (blue arrow) and few inflammatory cells (yellow arrow); and (**d**) periapical granuloma (detail), inflammatory infiltrates, and fibrous stroma with prominent fibroblasts (yellow arrow). Hematoxylin–eosin staining, ×400 magnification.

We classified the histological types of periapical granuloma into early, intermediate, and late stages based on the associated inflammatory cells (Figure 2a–d).

### 3.2. Immunohistochemical Findings

**Figure 3:**
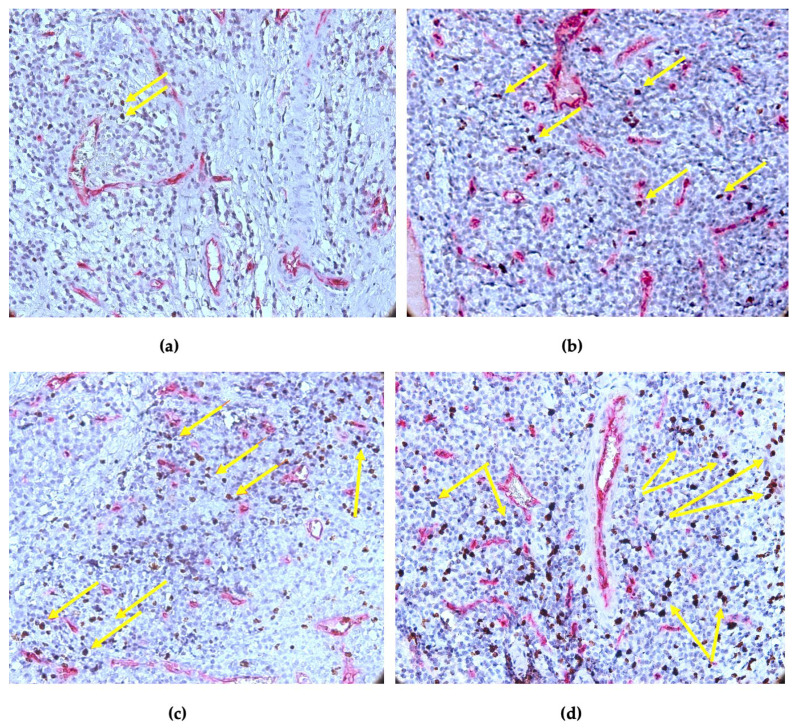
Ki-67 expression in periapical granulomas: (**a**) score 0 (1% positive nuclei, brown–yellow arrows); (**b**) score 1 (1–10% positive nuclei, brown–yellow arrows); (**c**) score 2 (>10–50% positive nuclei, brown–yellow arrows); and (**d**) score 3 (50% positive nuclei, brown–yellow arrows). Double CD34-Ki67 immunostaining, ×400 magnification.

During the immunohistochemical staining experiment, we observed the number of CD34-positive vessels and the number of Ki-67-positive cell nuclei. The positive expression of Ki-67 in the connective tissue resulted in a distinct nuclear brown staining that had a score of 0 to 3, as shown in Figure 3.

A positive reaction for the Ki-67 antibody was observed in 32 (86.5%) of the 35 cases of the periapical granulomas included in our study. A moderate expression (>10–50%) was the most frequently observed, as it was observed in 22 cases (59.5%). A light expression of Ki-67 (1–10%) was observed in 10 cases (27%), and in 3 cases (13.5%), Ki-67 expression was absent.

**Figure 4:**
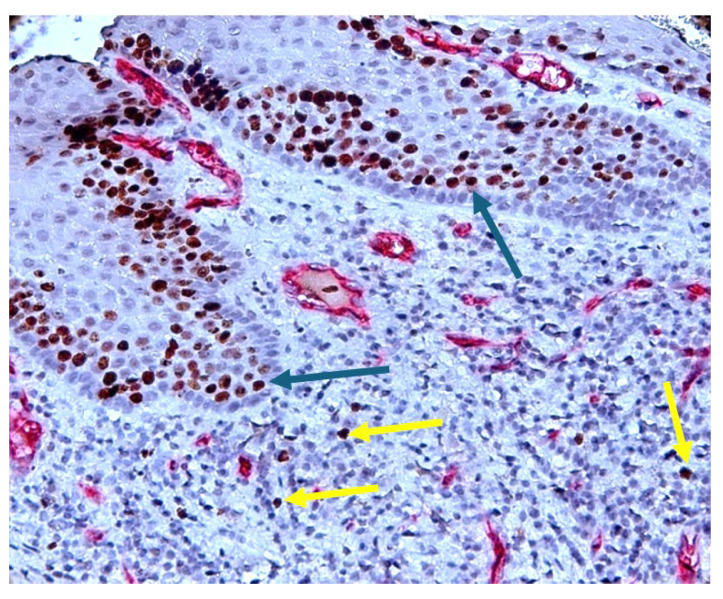
Stratified squamous epithelium with Ki-67-positive nuclei (brown, pointed to with blue arrow) in the basal area (positive control), a reduced number of Ki-67-positive nuclei in the area of the inflammatory infiltrate (brown, pointed to with the yellow arrows). CD34-Ki-67 double immunostaining, ×400 magnification.

As shown in Figure 4, we observed a high Ki-67 nuclear expression in the region of the inflammatory infiltrate, as well as in the epithelium’s basal layer.

The positive expression of CD34 in the vascular endothelium resulted in cytoplasmic red staining. We noticed CD34-positive vessels in all the cases included in this study. The most numerous examples were observed in periapical granulomas classified as being in the intermediate stage with mixed inflammatory infiltrates. The vessels present in the area of the inflammatory infiltrate were heterogeneous in terms of morphology and size. Most of the immunohistochemically identified vessels were medium or small in size, though only some showed lumen. Small vessels with a narrow lumen bordered by proliferating endothelial cells were also present. We noted the CD34-positive vessels that formed compartments in the area of the inflammatory infiltrate. Numerous inflammatory cells were present in these vascular compartments.

**Figure 5:**
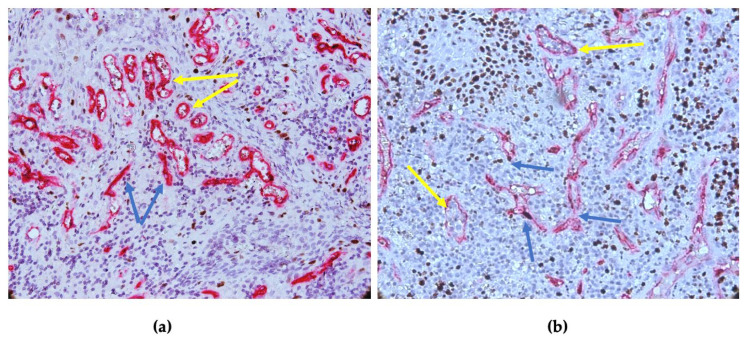
Vascular heterogeneity in periapical granulomas: (**a**) CD34-positive vessels varied in size and morphology, with both vessels without lumen (red–blue arrows) and small- and medium-sized vessels showing lumen (red–yellow arrows); and (**b**) CD34-Ki-67 co-expression at the level of the vascular endothelium, with vascular compartments. CD34-Ki-67 double immunostaining, ×400 magnification.

CD34 and Ki-67 co-expression was noted in the vascular endothelium, which allowed for the quantification of the vascular microdensity in relation to endothelial proliferation, as shown in Figure 5a,b.

We also noted that, in some cases, intussusception characterized by the presence of vessels had a wide lumen, with protrusions noted towards the endothelium lumen. All the above aspects suggest the activation of angiogenesis in periapical granulomas.

The proliferative activity of periapical granulomas is an important indicator for evaluating the progression and proliferation potential of the lesion and the pathological tissue. Positive Ki-67 expression, being a consequence of a chronic irritation, can be an indicator of the lesion’s evolution.

Thus, based on these results, our study is the only one to evaluate both Ki-67 and CD34 expression in periapical granulomas.

## 4. Discussion

Periapical granulomas are a consequence of a persistent bacterial infection localized in the roots of teeth, determining both the inflammatory response and the changes in the local environment triggered by the release of cytokines and growth factors [31]. They are histologically described as being rich granulation tissue, encapsulated by a fibrous membrane. Past studies discuss the cellular components of the granulation tissue, highlighting the presence of lymphocytes, monocytes, macrophages, and plasma cells resulting from chronic persistent inflammatory stimulus in the periapical space [32]. The reported humoral immune and cell-mediated reactions in periapical granulomas are involved in ongoing cellular proliferation [33]. The literature has reported these types of lesions in males and females [34]. In the present study, 51% of the samples belonged to males (18 patients), while the other studies reported a predominantly female population [35,36,37].

Comparing the prevalence between the different age groups, the literature reveals a higher incidence in the third and fourth decades of life [38]. However, there are studies that discuss the fact that inflammatory cysts are more commonly encountered in young adults, with this distribution most probably being determined by the oral and dental status of the studied population [39]. However, in the present research, the subjects had a median age of 43 years old. Regarding the localization of the periapical granulomas, the mandibular location was more common in our study, being similar to other existing data in the research field [37].

Studies have described the evolution of periapical inflammatory lesions as being directly dependent on the balance between cell proliferation and apoptosis [19]. Taking into consideration the fact that periapical granulomas are a response to bacterial stimuli that determine an inflammatory reaction, the release of inflammatory cytokines induces certain cellular stress that influences the immunoexpression of Ki-67 in periapical granulomas [22].

The Ki-67 antigen is a nuclear protein expressed by the cells that undergo a proliferating phase, with peak values in the phases G2 and M [40]. Taking this into consideration, researchers aimed to evaluate Ki-67 antibody expression in various malignancies [41]. Nevertheless, there are studies that focus on the presence of Ki-67 and PCNA in premalignant and malignant oral cavity lesions due to their implications for the proliferation process [42]. In addition, there is evidence related to the use of the immunoexpression of Ki-67 as a biological marker for the evaluation of a possible predisposition towards developing a cystic lesion [43]. The results of our study revealed positive Ki-67 immunoreactivity in the nuclei of the basal layers of the periapical granulomas compared to the low expression encountered in the nuclei of the inflammatory infiltrate. Indeed, similar results were reported by Sargozalei et al. [22]. In a study conducted by Slotweg et al. [44], it was noted that in the case of inflammatory periapical lesions, the expression of Ki-67 was higher in the basal layer compared to the odontogenic keratocysts, which exhibited a higher expression in the suprabasal layer. The results of the study performed by De Palma et al. [45] showed that the expression of Ki-67 in inflammatory keratocysts was higher compared to the non-inflammatory ones. These differences also influenced the different development pathways of these pathological entities [46]. The existing results show the importance of the odontogenic inflammatory entities and the evaluation of Ki-67 immunoexpression, offering a new perspective on the proliferative potential of a periapical lesion and, in some cases, its recurrence potential [46,47]. Chaturvedi et al. [47], in their study, identified the potential use of Ki-67 as a biomarker to evaluate the aggressiveness of benign odontogenic tumors. The results of their study indicated a positive correlation between the intensity of Ki-67-positive cells and the aggressive behaviors of the odontogenic tumor.

By targeting CD34 in periapical granulomas, we aimed to quantify angiogenesis in the pathological tissue. CD34 is defined as an adhesion molecule expressed in the endothelial and hematopoietic cells [48]. There are studies that show a positive correlation between CD34 and the intensity of the inflammatory infiltrate in pathological periapical tissue. In the present study, we reported similar results, identifying a higher immunoexpression of CD34 in the areas with a higher inflammatory infiltrate. In addition, by correlating the immunoexpression of CD34 with Ki-67, the results were positive, allowing us to identify a relationship between angiogenesis, the microvessel density, and the endothelial proliferation potential. The aggressive behavior of the periapical lesions was also linked to the intensity of CD34 expression, and the results of Mathiou et al. [49] describe the increases in the immunoexpression of CD34 and the microvessel density in the areas with an inflammatory infiltrate as increasing angiogenesis and influencing further development. Another study that focused on the presence of CD34 in periapical granulomas reported an increased expression of this molecule, determining the existence of an endothelial hyperplasia due to the increased angiogenesis [50].

The cellular and molecular mechanisms involved in granuloma progression and recurrence, particularly in terms of therapeutic interventions, could be used in future studies for the investigation of other osseous tumors like central giant cell granulomas. The etiology of this tumor type remains multifaceted and continues to be debated within the medical community, but early hypotheses have suggested an inflammatory, reactive reaction [51].

On the other hand, prophylaxis related to the occurrence of periapical granulomas must be studied further. Due to the implications of oral microbiota, the eubiotics administered to patients can help the local immune system of the oral cavity to stop the worsening of periapical injuries, as can the use of paraprobiotics (tyndallized probiotics) and postbiotics. Paraprobiotics are deactivated microbial cells that benefit the consumer without posing any health risks; they control the innate and adaptive immune systems, act as antagonists against pathogens, and have anti-inflammatory, antiproliferative, and antioxidant properties. Postbiotics, which comprise any material released or created by the metabolic activity of a microbe without including living bacteria themselves, should not be confused with probiotics and paraprobiotics [6]. Other factors that limit the formation and progression of a periapical granuloma can lead to good oral health, lowering the plaque index, ensuring the early detection and treatment of pulp inflammation and infection, and enabling the correct endodontic treatment of teeth with periapical symptoms.

Upon analyzing the expression of CD34 and Ki-67 in the samples, the results highlight the potential use of these proteins as biomarkers to evaluate the proliferative characteristics, inflammatory components, and future development of lesions.

One of the major limitations of this study is the number of samples. Our results should encourage new studies with larger samples to evaluate odontogenic periapical granulomas and improve the knowledge in this field. In addition, our findings and other potential studies could be the basis of antiangiogenic therapy.

## 5. Conclusions

Periapical granulomas are odontogenic pathologic entities that occur in response to chronic bacterial irritation. Pathological mechanisms and cellular interactions have an important influence on the evolution of lesions. In the present study, we identified the overexpression of Ki-67 and CD34 and calculated the Ki-67 index to evaluate and correlate the proliferation potential and angiogenesis in the presence of an inflammatory infiltrate. These findings suggest that the persistence of the inflammatory environment directly influences Ki-67 and CD34 expression, sustaining the proliferative capacity of the cells and abnormal angiogenesis.

## Figures and Tables

**Table 1:** Patient baseline characteristics.

	*N* = 35
Age	43 (36, 50)
Sex	
M	18 (51%)
F	17 (49%)
Smoking status	
Non-smoker	25 (71%)
Smoker	10 (29%)
Daily cigarette consumption	20 (16, 3–20)
Extracted tooth	
1	29 (83%)
2	5 (14%)
3	1 (3%)

## Data Availability

The data presented in this study are available on request from the corresponding author. The data are not publicly available due to restrictions related to the privacy of the funding protocol.
